# From observational to dynamic genetics

**DOI:** 10.3389/fgene.2014.00006

**Published:** 2014-01-21

**Authors:** Claire M. A. Haworth, Oliver S. P. Davis

**Affiliations:** ^1^Department of Psychology, University of WarwickCoventry, UK; ^2^Department of Genetics, Evolution and Environment, UCL Genetics Institute, University College LondonLondon, UK

**Keywords:** intervention, gene-environment interaction, dynamic genetics, Twins, heritability

## Abstract

Twin and family studies have shown that most traits are at least moderately heritable. But what are the implications of finding genetic influence for the design of intervention and prevention programs? For complex traits, heritability does not mean immutability, and research has shown that genetic influences can change with age, context, and in response to behavioral and drug interventions. The most significant implications for intervention will come when we move from observational genetics to investigating dynamic genetics, including genetically sensitive interventions. Future interventions should be designed to overcome genetic risk and draw upon genetic strengths by changing the environment.

## INTRODUCTION

Genetically sensitive research has a history of being misinterpreted and misunderstood. In the early days of behavioral genetics there were extreme views of genetic determinism versus environmental determinism (Editorial, 2012), or *Nature versus Nurture*. Behavioral genetics has now matured as a field, and empirical data have highlighted the importance of both genes and environments in creating individual differences in behavior. Modern behavioral genetics investigates the complex interplay between probabilistic genetic and environmental risks (van Dongen et al., 2012; Plomin et al., 2013). Nevertheless, there remains a bias toward biological findings meaning immutability, whereas environments are potential “modifiable risk factors.” In this perspective we consider the implications of finding genetic influence for intervention and prevention programs. Is a trait that is 20% heritable easier to change than a trait that is 80% heritable? Our intention is to demonstrate the dynamic nature of both genetic and environmental influences during development and in response to the environment, and to show how improving our understanding of dynamic genetics could help us design better, more effective interventions.

## HERITABILITY CAN CHANGE

The meaning of heritability – the proportion of variation in a population accounted for by genetic variation – is difficult to grasp (Visscher et al., 2008). First of all, the focus of heritability is on what makes people different from each other, so although it is obvious that genes are involved in every aspect of growth and development of a human body, heritability is only concerned with the DNA that varies between people and the extent to which that genetic variation leads to differences between us. This leads, in part, to an important misunderstanding about what heritability means for a single individual. For example, if we use the results we have for height, we can say that heritability estimates for height are around 80% (Silventoinen et al., 2003). However, this does not mean that 80% of one individual’s height is explained by genes they inherited from their parents, and that the remaining 20% of their height is due to environments like their diet. What it does mean is that of the variation we see between people in height, 80% of that variation is explained by genetic differences in the population. Like a sample mean or variance, heritability is a population-level statistic, and does not imply anything about how “genetic” a trait is for an individual.

A related misunderstanding about genetic influence is that because our DNA sequence does not change, then once we know how important genes are, they will always be that important. This assumption is wrong – many studies have now shown that genetic influences can change. Here we provide examples relating to changes that occur developmentally, as well as changes that occur in response to context.

### DEVELOPMENTAL CHANGES IN HERITABILITY

With the exception of localized mutations brought about by exposure to, for example, certain chemicals or ionizing radiation, our DNA sequence remains the same throughout our lives. On the other hand, environmental experiences accumulate, so it seems reasonable to assume that environmental influences will become proportionately more important as we grow up. However, heritability has been shown to *increase* *developmentally* for various traits (Bergen et al., 2007) including cognitive abilities (Haworth et al., 2010), body mass index (BMI; Haworth et al., 2008), and anxiety (Bergen et al., 2007). For cognition, a mega-analysis combining six twin studies comprising 11,000 pairs of twins, found that the heritability of intelligence increased from 41% in early childhood, to 55% in adolescence and 66% in young adulthood (Haworth et al., 2010). This finding is repeated in longitudinal studies that follow the same individuals through development (Davis et al., 2009). One likely mechanism is the interplay between genes and environments, and in particular the increasing role of active gene-environment correlation as we grow up (Rice et al., 2003; Briley and Tucker-Drob, 2013). Active gene-environment correlation describes how our genes influence the way we experience the environment, leading us to select or seek out environmental experiences that are correlated with our genetic propensities. In the case of cognitive abilities, this may manifest, for example, as young people selecting peers who are also motivated to do well at school, or seeking out cognitive stimulation via books, science clubs, and museum trips. Selecting these experiences will lead to additional experiences, and these upward (or downward) spirals of exposure driven by genetic propensities will serve to make genetic factors more important. As we get older we attain more control over the experiences we select, which could explain the developmental appearance of these increases in heritability. Of course, there are other possible explanations for changes to heritability developmentally, which might include reciprocal changes in the variability of environmental experiences, or changes in the environmental experiences that influence the trait. However, these simpler alternatives can often be ruled out by parallel analyses of unstandardized variance components.

Heritability does not increase for all traits. For example, attention deficit hyperactivity disorder (ADHD) and autism spectrum disorders typically have high heritability in childhood, which remains high in adolescence. There is even some indication that the heritability of ADHD in adulthood may be lower than that in childhood (Boomsma et al., 2010; although see Larsson et al., 2013, for discussion of rater effects), and another exception to the default of increasing heritability for cognitive abilities is school science performance, which shows significant decreases in genetic influence between ages 9 and 12 years (Haworth et al., 2009). An important question is whether changes in heritability are reflected by changes in DNA associations developmentally. One phenotype that shows developmental changes in heritability and has well replicated DNA associations is BMI (Frayling et al., 2007). For BMI, changes in heritability are paralleled at the molecular level with changes in the effect size of the FTO gene (Haworth et al., 2008). As more DNA associations are found and replicated, we predict that more of these age-dependent associations will be uncovered. These findings suggest that the changes in heritability with age are not an idiosyncrasy of the twin design; these changes are also reflected at the molecular level, showing that the effect of DNA variants on a phenotype can change, even though the DNA sequence itself remains the same.

### CONTEXTUAL CHANGES IN HERITABILITY

**FIGURE 1:**
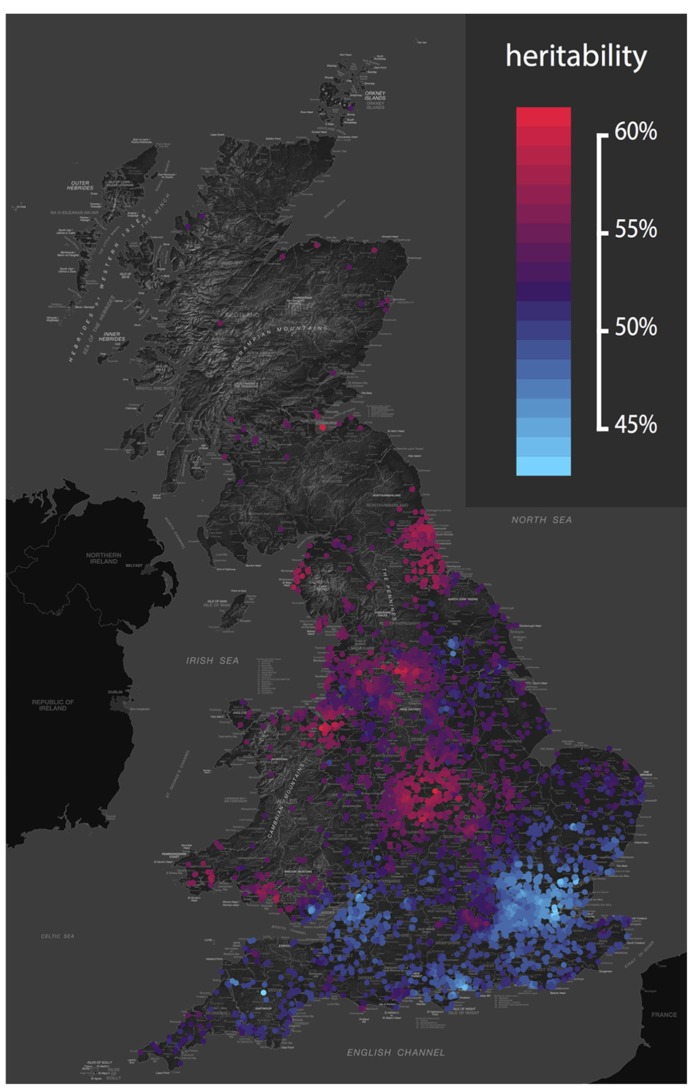
**Geographical variation in the heritability of antisocial behavior.** The genetic and environmental etiology of many traits varies across the UK. This map shows the heritability of classroom antisocial behavior from an analysis of over 6,000 pairs of UK twins (Davis et al., 2012). The color scale ranges from red (high) to blue (low), revealing relatively low heritability in the south, and relatively high heritability in the north. This study was conducted in a homogeneous UK population, so these heritability hotspots are areas in which the environmental context acts to *draw out* genetic differences between people. For example, genetic influences on hay fever would not be noticeable in a region without wind-pollinated crops, yet in an area with airborne pollen, genetic propensities to hay fever will be revealed. Again, analysis of raw variance components tells us that these geographical differences in genetic influence cannot be explained by simple reciprocal differences in the variability of the local environment. Genetic and environmental maps of the UK for 45 childhood phenotypes are available from http://bit.ly/tedsgeo.

Heritability can also change in response to the *environmental* *context*. Gene-environment interaction results in differences in heritability based on environmental exposure. For example, heritability of depression, wellbeing and drinking behavior varies depending on marital status (e.g., Nes et al., 2010). And genetic influences on adolescent depression are greater in those experiencing more life events, and harsh maternal discipline (Lau and Eley, 2008). Again, similar effects are seen at the molecular level (Caspi et al., 2010). We recently developed a new approach to twin data that assesses whether the importance of genes and environments vary based on geographical location (Davis et al., 2012). The approach allowed us to identify genetic and environmental “hotspots” – areas where genetic variation or environmental variation accounts for more variance – for childhood traits including language and antisocial behavior (see **Figure 1**).

The crucial point is that heritability is a context-specific statistic, as are DNA-phenotype associations. Genetic influence can change in relation to the developmental process, and in response to environmental changes. Acknowledging the dynamic properties of genetic and environmental influence on complex traits has significant implications for the way we think about interventions and behavior change. Interventions could change the importance of genetic (and environmental) influence because they alter the “exposome”: the environmental context of our genomes (Wild, 2012).

## IS IT POSSIBLE TO CHANGE HERITABLE TRAITS?

Another angle is to ask whether it is possible to change heritable traits. The reifying of biological and genetic influence over the impact of social, cultural, and environmental factors seems to be related to the idea that biology is harder to change. We agree that the likelihood of genetic engineering for complex traits is very slim. However, it will still be possible to mitigate genetic risk factors through environmental interventions. These “environmental” interventions include everything other than altering DNA sequence. So they could include psychological therapies, surgery, and drug therapies, as well as universal interventions such as education and social policy changes. None of these alter DNA sequence, but they may affect the relationship between genotype and phenotype, which can change in different contexts, as we have already seen.

The classic example in genetics is Phenylketonuria (PKU), which went from being 100% heritable to being 0% heritable. Individuals with PKU are born with a defective gene for the enzyme that breaks down phenylalanine, which leads to increased blood levels of this amino acid and concomitant abnormal brain development and learning difficulties. Severe mental retardation can develop within a year if left untreated (Widaman, 2009). Understanding the genetic and environmental causes, and in particular the way in which the two interacted, allowed the development of a very effective environmental intervention. People in many countries are now screened for PKU at birth. Those carrying the gene mutation are treated by eliminating their dietary exposure to phenylalanine [National Institutes of Health (NIH), 2000]. Effective treatment requires strict adherence to the diet, which is difficult given the pervasiveness of the amino acid in food. Nevertheless, it is possible to overcome this genetic disease through an entirely environmental intervention.

What about traits with more complex etiology that are affected by many genes of small effect and many environmental exposures? Medicine provides some good examples of overcoming genetic influence, even for highly complex and highly heritable traits. Obesity heritability estimates range from 40 to 90% (Elks et al., 2012). One intervention for severe cases of obesity is bariatric surgery, with a mean reduction of 14.20 BMI points, as well as complete diabetes resolution in 77% of patients (Buchwald et al., 2004). Another medical intervention with similar successes is using metformin and lifestyle changes for the treatment of diabetes (Knowler et al., 2009). There are examples within psychology and psychiatry too, including increases in intelligence following adoption (Duyme et al., 1999), cognitive behavioral therapy for mild to moderate depression (NICE, 2004), and parenting interventions for antisocial behavior (Scott et al., 2009).

**FIGURE 2:**
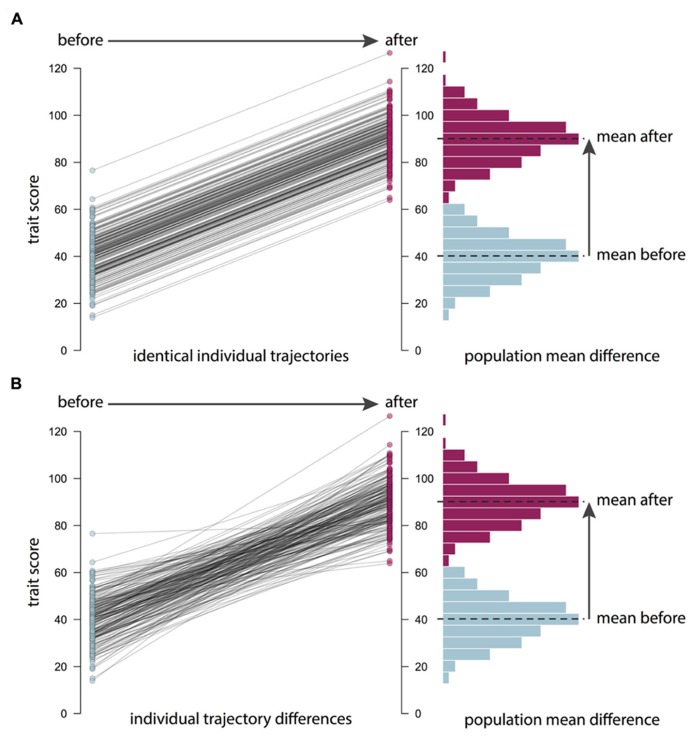
**Mean differences versus individual differences.** Changes in the population or sample mean do not necessarily imply changes in individual differences. For example, in **(A)**, an intervention or another environmental change leads to an increase in the mean trait score in a population of 200 individuals: the whole distribution is shifted, as shown in the histogram on the right. However, the plot on the left shows that the trajectories of the individuals in the population are identical, so individual differences statistics, such as the heritability of the trait, are unaffected. This is the situation with the recent increase in body mass index (BMI) in many countries: even though there has been a mean increase for environmental reasons, BMI remains highly heritable. In contrast **(B)** shows a situation where individuals follow different trajectories in response to an intervention: while some individuals show large increases in the trait, others are unaffected, perhaps because of genetic differences between them. In this case, the change in the population mean may be accompanied by a change in heritability. Going beyond assessing the mean effects of an intervention to explore the genetic and environmental etiology of intervention response will help us to understand how and why an intervention is (or is not) working.

It is important to remember that the causes of individual differences, or population variance in a trait, may be unrelated to the causes of changes in the population mean (see **Figure 2**). For example, average BMI worldwide has been increasing over the last 30 years (Finucane et al., 2011). Genetic factors are unlikely to explain these recent changes in BMI, because the human genome does not change so fast. What have changed are our environmental exposures and experiences. However, there is still variance in BMI. That is, there is still a distribution of weights in the population from low to high. Heritability is concerned with what causes these individual differences between people, regardless of the population mean. The importance of genetic factors for creating these individual differences in BMI has remained stable even though mean BMI has increased (Wardle et al., 2008). For similar reasons, heritability estimates do not necessarily tell us about how easy it will be to change the mean levels of a trait. And conversely, being able to change a trait, for example through training or practice, tells us nothing about the importance of genes in explaining individual differences in the population. It follows that the success of an intervention will likely be unrelated to how highly heritable a trait is. In observational genetics, heritability statistics only tell us about “what is”; they tell us absolutely nothing about “what could be” when we introduce a novel environmental intervention.

## GENETICALLY SENSITIVE INTERVENTIONS

Finding heritability, whether small or large should not be an obstacle for attempting interventions. We have seen that even a highly heritable trait can be changed by using an effective environmental intervention. Nevertheless, we believe that there is a place for genetics in intervention, and that is in understanding individual differences and revealing mechanisms, both of which could lead to interventions that are more effective and longer lasting.

Pharmacogenomics is the traditional way in which genetics has been incorporated into intervention designs (Uher, 2011). The aim of pharmacogenetics is to identify DNA variants that could predict drug response, and eventually allow the tailoring of prescription to individual genetic profiles. Genetics is now being incorporated into behavioral interventions as well (Plomin and Haworth, 2010), including differential genetic susceptibility to parenting and cognitive behavioral therapy interventions (Bakermans-Kranenburg et al., 2008; Van IJzendoorn et al., 2011; Eley et al., 2012).

However, as with other molecular genetic investigations, the stumbling block is in identifying DNA variants that are reliably associated with outcome or treatment response (McCarthy et al., 2008). So far, these behavioral intervention studies have relied upon candidate genes, rather than considering millions of DNA markers across the genome, which is the current approach in other areas of molecular genetics. In addition, the intensive and expensive study designs have limited the sample sizes available for well-powered genetic analyses. An alternative method is to use genetically sensitive designs that allow the investigation of genetic and environmental influence without needing to know which specific genetic variants are involved (Visscher et al., 2008; Haworth and Plomin, 2010). Using designs such as twin, family, and adoption studies, as well as estimating genetic variance from genome-wide genotype data, will allow us to understand more about how dynamic genetics works. Although these designs do not give us purchase on the specific DNA variations involved, they can provide information about how important genetic factors are in explaining individual differences in treatment response, and whether the same genes (and environments) are active before and after the intervention. These results could lead to changes in the way we incorporate DNA data into intervention studies. Candidate genes used in intervention studies are typically selected based on a previous association with the trait of interest, but genetic influences on baseline and on intervention response are not necessarily the same. If twin intervention studies uncover what happens to genetic influences during an intervention (including whether it is the same genes at play for both baseline and response), then we can use this knowledge to guide the selection of candidate genes for DNA intervention studies. Such twin studies will also quantify the role of genetic factors in explaining differential susceptibility to interventions. There are a variety of genetically sensitive intervention designs, as discussed previously in a special issue of *Perspectives on Psychological Science* (Reiss, 2010). The key contribution that these designs will make will be in understanding individual differences in intervention response, a trait typically treated as the error term in traditional intervention designs that focus on mean changes. These genetic approaches parallel recent studies in intervention science that have considered other individual differences such as personality as possible moderators of intervention effects (e.g., Stoltz et al., 2013). Understanding why an intervention works better for some people will help us to understand why the intervention works at all, as well as whether it will be beneficial to think of personalizing interventions using genetic (e.g., family history) and environmental information available at baseline. We believe that considerable advances can be made by capitalizing on the dynamic nature of genetic and environmental influences and by combining this with a focus on individual differences as well as mean differences in intervention designs.

## THE FUTURE OF DYNAMIC GENETICS

As we move from observational to dynamic genetics we will encounter gaps in our knowledge of how genetic and environmental influences have their effect. As a starting point, we briefly outline some initial questions for the future of dynamic genetics.

First, we know very little about whether knowing *what is* can predict *what could be. *More specifically, we need to test using genetically sensitive designs whether knowing about genetic and environmental influences on a trait does help us to conduct better interventions. Second, we need to investigate the concept of a *genetic set point. *Why do individuals often rebound to their pre-intervention state? Third, what is a modifiable risk factor? Given this new perspective on genetics as a dynamic influence, shouldn’t we stop referring to genetics as an unmodifiable risk factor? Fourth, do some traits emerge so early in development that we do not have time to overcome genetic risk? At what point has an individual’s pathway been determined? Is there a critical period where treatment is most effective? A related point is whether interventions should be targeted in the early years when heritability is (often) lower. We do not believe that this necessarily follows from heritability results: heritability should not be used as the sole reason for the timing of interventions. Fifth, combining genetics and prevention requires prediction, but at present molecular genetic information provides very little predictive power. Are there other ways in which we can use genetic risk without using DNA? For example, the concept of dynamic genetics might re-ignite interest in using family history to guide intervention programs, like the British Autism Study of Infant Siblings (Elsabbagh and Johnson, 2010). Sixth, we need to study positive traits and resilience because understanding the causes of healthy outcomes should help us to design interventions that push more people toward health and flourishing. Some gene-environment interactions can lead to positive outcomes, and these are just as important to study as those that lead to negative outcomes. Finally, we need to advance our understanding of the biological embedding of experiences and interventions. How do behavioral interventions get under the skin and into the brain? A possible mechanism is via epigenetic processes that provide a pathway between environmental experiences and changes in gene expression.

## PERSPECTIVE

This perspective on the dynamic nature of genetic and environmental influences in creating individual differences tells us that finding significant baseline heritability is not a barrier to environmental or behavioral intervention. However, although cures do not necessarily have to fix the underlying cause, it is likely that interventions that target causal pathways will be more effective and longer lasting. For this reason, understanding the underlying genetic and environmental etiology of individual differences can lead to improvements in the design and targeting of interventions.

## Conflict of Interest Statement

The authors declare that the research was conducted in the absence of any commercial or financial relationships that could be construed as a potential conflict of interest.
